# Overexpression of the MRE11-RAD50-NBS1 (MRN) complex in rectal cancer correlates with poor response to neoadjuvant radiotherapy and prognosis

**DOI:** 10.1186/s12885-018-4776-9

**Published:** 2018-09-03

**Authors:** Vincent Ho, Liping Chung, Amandeep Singh, Vivienne Lea, Askar Abubakar, Stephanie H. Lim, Weng Ng, Mark Lee, Paul de Souza, Joo-Shik Shin, Cheok Soon Lee

**Affiliations:** 1 MBBS FRACP, School of Medicine, Western Sydney University, Locked Bag 1797, Penrith, NSW 2751 Australia; 2 Ingham Institute for Applied Medical Research, Liverpool, NSW 2170 Australia; 3 Department of Anatomical Pathology, Liverpool Hospital, Liverpool, NSW 2170 Australia; 4 Macarthur Cancer Therapy Centre, Campbelltown Hospital, Campbelltown, NSW 2560 Australia; 5 Department of Medical Oncology, Liverpool Hospital, Liverpool, NSW 2170 Australia; 6 Department of Radiation Oncology, Liverpool Hospital, Liverpool, NSW 2170 Australia; 7 Discipline of Medical Oncology, School of Medicine, Western Sydney University, Liverpool, NSW 2170 Australia; 8 Tissue Pathology and Diagnostic Oncology, Royal Prince Alfred Hospital, Camperdown, NSW 2050 Australia; 9 Discipline of Pathology, School of Medicine, Western Sydney University, Campbelltown, NSW 2560 Australia; 10 Faculty of Medicine, South Western Sydney Clinical School, The University of New South Wales, Liverpool, NSW 2170 Australia

**Keywords:** DNA damage response, MRE11-RAD50-NBS1 complex, Rectal cancer, Prognosis, Biomarkers, Neoadjuvant radiotherapy

## Abstract

**Background:**

The MRE11/RAD50/NBS1 (MRN) complex plays an essential role in detecting and repairing double-stranded breaks, and thus the potential roles of MRE11, RAD50 and NBS1 proteins in the pathogenesis of various cancers is the subject of investigation. This study was aimed at assessing the three-protein panel of MRN complex subunits as a potential radiosensitivity marker and evaluating the prognostic and clinicopathological implications of MRN expression in rectal cancer.

**Methods:**

Samples from 265 rectal cancer patients treated with surgery and adjuvant chemoradiotherapy, including samples from 55 patients who were treated with neoadjuvant radiotherapy between 2000 and 2011, were analyzed. Expression of MRN complex proteins in tissue samples was determined by immunohistochemistry. Univariate and multivariate analyses were carried out to identify clinicopathological characteristics that are associated with the MRN three-protein panel expression in rectal cancer samples.

**Results:**

In Kaplan–Meier survival analyses, we found that high level expression of MRN complex proteins in postoperative samples was associated with poor disease-free (*p* = 0.021) and overall (*P* = 0.002) survival. Interestingly, high MRN expression also correlated with poor disease-free (*P* = 0.047) and overall (*P* = 0.024) survival in the neoadjuvant radiotherapy subgroup. In multivariate analysis, combined MRN expression (hazard ratio = 2.114, 95% confidence interval 1.096–4.078, *P* = 0.026) and perineural invasion (hazard ratio = 2.160, 95% confidence interval 1.209–3.859, *P* = 0.009) were significantly associated with a worse disease-free survival.

**Conclusions:**

Expression levels of MRN complex proteins significantly predict disease-free survival in rectal cancer patients, including those treated with neoadjuvant radiotherapy, and may have value in the management of these patients.

**Electronic supplementary material:**

The online version of this article (10.1186/s12885-018-4776-9) contains supplementary material, which is available to authorized users.

## Background

Colorectal cancer (CRC) is one of the most prevalent malignancies. Although CRC patient mortality rates are decreasing because of improved screening and treatment methods, CRC remains the third most commonly diagnosed cancer in the United States [1] and the third most important cause of cancer-related death globally [2]. Colonic and rectal cancers are often combined as a single entity in many studies but they differ in their metastatic pattern, drug response, and optimal treatment methods [3]. Rectal cancer patients experience poorer survival outcomes than colon cancer patients because resection is more difficult [4]; therefore, selection of appropriate treatment is especially important for rectal cancer patients.

Neoadjuvant (i.e., preoperative) radiotherapy or chemoradiation is routinely used to treat patients with rectal cancer to improve surgical outcomes [3]. Many studies have found that this therapy improves overall survival (OS) and reduce recurrence [5, 6]. Meta-analyses have shown that neoadjuvant radiotherapy improves local control [7], prevents recurrence [8], and reduces mortality [9], but the effect on a given outcome varies greatly between studies. This inconsistency is due in part to the large variation in outcomes between patients. Only 10–30% of patients show a complete response to preoperative chemoradiation, and 70% show a decrease in the tumor stage [10, 11]. Neoadjuvant radiotherapy also has a significant risk of adverse effects in rectal cancer patients, especially gastrointestinal disorders such as bowel obstruction, abdominal pain, and nausea [12]. Although these adverse effects have become less prevalent as irradiation techniques have improved [13], it remains important to select patients carefully for radiotherapy to avoid unnecessary side effects.

Many studies have sought biomarkers that would predict the patient’s response to radiotherapy or chemoradiotherapy, including imaging findings, gene mutations, and expression levels of mRNAs and proteins [14]. The radiosensitivity index (RSI) is a 10-gene signature that predicts the response to radiotherapy in cell lines [15], and has been shown to predict OS in glioblastoma patients [16]. A radioresistance (RadR) score calculated based on expression of 13 genes was associated with recurrence in head and neck squamous cell carcinoma patients treated with adjuvant radiotherapy [17]. More than 40 potential molecular biomarkers have been assessed for their ability to predict outcomes in rectal cancer patients receiving neoadjuvant therapy, often with conflicting results [14]. Therefore, there is still a great need for simple, accurate biomarkers that will predict rectal cancer patient outcomes.

DNA damage repair has been described as a “double-edged sword” in cancer [18]. Defective repair can lead to genome instability and promote cancer formation. Deficiencies in the mismatch repair (MMR) pathway, for example, cause a hypermutability phenotype known as microsatellite instability (MSI). This can lead to overall genetic instability and mutations in many other genes that promote cancer development and progression [19]. MSI is found in approximately 15% of rectal cancers [20]. Conversely, DNA damage is also the mechanism by which radiotherapy and some chemotherapy treatments cause cancer cell death. Thus, cancer cells that can efficiently repair the damage may become resistant to such therapies. The link between DNA damage repair and radiotherapy makes DNA damage-related proteins attractive targets for developing new therapies and for identifying markers of sensitivity to existing therapies. For example, the levels of phosphorylated DNA damage repair related proteins ATM and γH2AX have been identified as biomarkers for radiosensitivity to ^12^C^6+^ radiation in various tumor cell lines [21].

Radiotherapy causes double-stranded breaks (DSBs) in DNA. The highly conserved MRN complex comprises the MRE11, RAD50, and NBS1 proteins, and is one of the first factors to sense and bind DSBs. The MRN complex can physically tether DNA ends together, and also plays an enzymatic role in DNA repair via the nuclease activity of MRE11 [22]. The cell cycle checkpoint kinase ATM is recruited to DSBs and activated with the help of MRN, and ATM then phosphorylates all three MRN subunits, demonstrating a role of the complex in cell cycle progression following DNA damage [23]. These roles of the MRN complex led us to hypothesize that tumors deficient in the MRN complex may be more sensitive to the DNA-damaging effects of radiotherapy. We have previously shown that the combined expression of two protein markers of MRE11 and ATM may be predictive of patient outcomes in rectal cancer [24]. We have also found that postoperative expression of RAD50 correlates to patient outcomes in rectal cancer [25]. In the current paper, we have extended our studies to include NBS1 and investigated whether the combined expression of the MRN complex proteins MRE11-RAD50-NBS1 may be superior in predicting patient outcomes after radiotherapy, and therefore useful for selecting the patients who would benefit from preoperative radiotherapy.

## Methods

### Patients

The study was conducted with the approval of the South Western Sydney Local Health District Human Research Ethics Committee (HREC Reference: HREC/14/LPOOL/186; project number 14/103). Surgical specimens were collected from 265 patients who were treated with chemoradiotherapy or neoadjuvant radiotherapy followed by surgery for rectal cancer during the period 2000–2011. Patients were treated with either a 50.4 Gy dose administered over 28 fractions or a 25 Gy dose of radiotherapy administered over five treatment fractions; the former also received 5-fluorouracil-based chemotherapy. Surgery comprised of anterior or abdominoperineal resection, with total mesorectal excision. Follow-up included clinic visits, blood tests, colonoscopy, and imaging which were done at the discretion of the treating specialist.

### Response and outcomes of interest

Short-term response to neoadjuvant radiotherapy was measured by tumor regression grade (TRG) according to the 7th edition of the American Joint Committee on Cancer manual [26]. Variables included pathological TNM stage, histological grade, age, sex, vascular invasion, presence of tumor-infiltrating lymphocytes, perineural invasion and treatment modality. TRG was excluded as a variable because the sample set of responders (6/55, 10.9%) was too small for meaningful statistical analysis. Parameters for long-term outcomes were disease-free survival (DFS; time from diagnosis to first recurrence) and OS (time from diagnosis to last follow-up or death) (both DFS and OS determined by Kaplan–Meir analysis).

### Sample preparation and tissue microarrays

Preparation of tissue microarray (TMA) slides from archival tissue samples of pre- and post-operative rectal cancer tissues from these patients has been described [25]. Tissue samples for analysis were obtained from five sites (two samples per site): tumor center (TC); invasive edge at tumor periphery (TP); adjacent normal mucosa; nonadjacent normal mucosa; involved lymph nodes. Representative areas of tumor and normal tissue were identified by microscopic analysis of H&E stained sections and used to prepare TMA slides as previously reported (REF) using a Beecher Manual Tissue Microarrayer (Beecher Instruments Inc., Sun Prairie, WI, USA).

### Immunohistochemical analysis

For immunohistochemistry, deparaffinization and antigen retrieval were performed as described [25]. The TMA slides were then incubated with mouse monoclonal primary antibodies including anti-RAD50 [13B3/2C6] (1:400 dilution, Abcam #ab89; Cambridge, UK), anti-MRE11 (1:600 dilution, Abcam #ab214) and anti-NBS1 (1:800 dilution, Novus Bioscience, NBP1–06609, Littleton, CO, USA) antibodies for 60 min at room temperature. Immunostaining of these samples for the mismatch repair proteins MLH1, MSH2, MSH6 and PMS2 has been described [25].

Samples were scored by two pathologists independently. Expression of the 3 MRN proteins was calculated as the product of intensity of staining and percent positive cells to produce a weighted score ranging from 0 to 12 as previously described [24]. Samples were categorized into a low (score range: 0–< 6) or high (score range: 6–12) expression group. Assessment for the mismatch repair proteins was based on positive or negative staining for MLH1, MSH2, MSH6 and PMS2 irrespective of the proportion of cells stained.

### Statistical analysis

SPSS for Windows 22.0 (IBM Corporation, Armonk, NY, USA) was used for statistical analysis. Survival analysis was conducted both on the entire cohort and, separately, on patients who received preoperative radiotherapy. MRE11, RAD50 and NBS1 expression were compared and combined by binary logistic regression as described previously [27]. Univariate and multivariate analyses of the combined expression of the three proteins at the TC and TP were performed using Kaplan–Meier curves and Cox’s proportional hazards survival modeling. Covariates were sex, age, TNM stage, histological grade, vascular invasion, perineural invasion, chemotherapy, and radiotherapy. Univariate analysis was performed using the Mann–Whitney U test. *p* < 0.05 was considered statistically significant.

## Results

### Study population

A total of 265 patients were included in this study; characteristics of the studied cohort are listed in Table 1. This cohort included 176 (66.4%) males, 89 (33.6%) females. The median age was 71 years (range: 35–100 years). All patients were followed for a median period of 3.16 years (range: 0–12.6 years) with a median time to death after surgery of 2.5 years (range 0–11.1 years). A cohort of 77 patients (31.3%) were treated with radiotherapy, with the majority of these (55 patients; 71.4%) having received preoperative therapy.

**Table 1:** Patient characteristics

	All Patients n (%)	Preoperative Radiotherapy Group
Total, n	265	55
Age (median)	71	66
Sex		
Male	176 (66.4)	37 (67.3)
Female	89 (33.6)	18 (32.7)
Tumor stage		
T1–2	87/260 (33.4)	17/55 (30.9)
T3–4	173/260 (66.6)	38/55 (69.1)
Node stage		
N0	140/259 (54.1)	29/55 (52.7)
N1–2	119/259 (45.9)	26/55 (47.3)
Metastasis stage		
M0	223/240 (92.9)	53/54 (98.1)
M1	17/240 (7.1)	1/54 (1.9)
Grade		
1–2	245/265 (92.5)	51/55 (92.7)
3	20/265 (7.5)	4/55 (7.3)
Vascular invasion		
Absent	201/263 (76.4)	47/55 (85.5)
Present	62/263 (23.6)	8/55 (14.5)
Perineural invasion		
Absent	220/263 (83.7)	41/55 (74.5)
Present	43/263 (16.3)	14/55 (25.5)
Radiotherapy		
Total	77/246 (31.3)	–
Neoadjuvant	55/77 (71.4)	–
Adjuvant	22/77 (28.6)	0/55 (0)
Recurrence		
Absent	131/213 (61.5)	25/46 (54.3)
Present	82/213 (28.5)	21/46 (45.7)
Tumor regression grade		
0–1 (good response)	–	6/55 (10.9)
2–3 (poor response)	–	49/55 (89.1)

### Establishment of a putative biomarker panel of MRN complex proteins

Expression levels of MRE11, RAD50 and NBS1 proteins in the TC were tested using a forward and reverse binary logistic regression analysis in an immunohistochemical scoring data set from 262 tumor samples and 258 normal tissues. The average receiver operator characteristic area under the curve (ROC-AUC) in the final biomarker model was 0.870 for the combined expression of the three MRN proteins. The expression of MRE11, RAD50 and NBS1 proteins in the TP (tumor, *n* = 261; normal, *n* = 258) were also tested, resulting in an average ROC-AUC of 0.862. For the MRN combined panel, the sensitivity was 89.0% and specificity was 77.2% in the TC, and was 78.2% and 77.6%, respectively, in the TP. Overall biomarker accuracy when measured in TC was 83.1% and when measured in TP was 77.9% (Table 2).

**Table 2:** Performance of the MRN three proteins combined classification models

Model	Tumor	Normal	Sensitivity (%)	Specificity (%)	Overall (%)	ROC-AUC
Combined TC^a^	262	258	89.0	77.2	83.1	0.870
Combined TP^b^	261	258	78.2	77.6	77.9	0.862

^a^Tumor center; ^b^Tumor periphery

### Association between the MRN combined expression and clinicopathological features

**Fig. 1:**
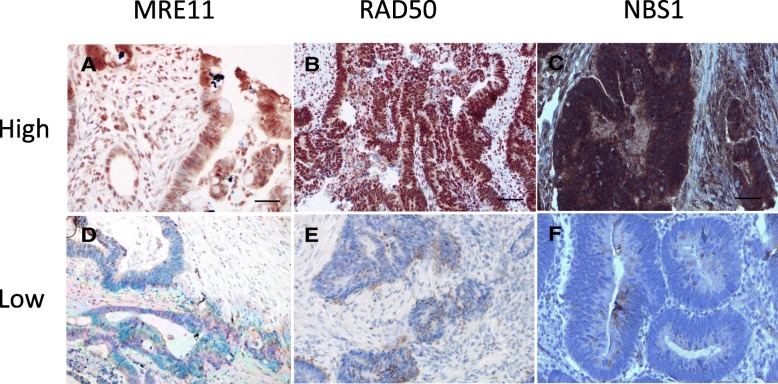
Immunohistochemical staining of MRE11, RAD50 and NBS1 proteins. Staining for each protein was scored as high or low as described in the Methods section. Representative examples of typical nuclear staining of MRE11 (**a**), RAD50 (**b**), and NBS1(**c**) scored as high expression in tumor cells. Correspondingly, examples of those scored as low expression for MRE11 (**d**), RAD50 (**e**), and NBS1 (**f**) are shown (40× magnification)

We investigated the association between the three-protein combined expression level and clinicopathological characteristics as summarized in Table 3. Representative immunohistochemical staining of high and low MRN combined three-protein expression in rectal cancer tissues is shown in Fig. 1. High MRN complex protein expression levels in the TC were significantly associated with histological tumor stage (*P* = 0.009). In a comparison of patients with low or high expression of MRN complex proteins, we did not observe significant differences in age, sex, lymph node involvement, metastasis, vascular invasion, or perineural invasion. Interestingly, we found that expression levels of MRN complex proteins were also significantly associated with preoperative neoadjuvant therapy (*P* = 0.021), with patients receiving neoadjuvant therapy more often having low combined MRN expression.

**Table 3:** Associations between combined MRN expression in the tumor center or periphery and clinicohistopathological data

	Tumor Center			Tumor Periphery		
	Low (%)	High (%)	*P* value	Low (%)	High (%)	*P* value
Sex						
Male	62.5	66.8	0.567	80.0	64.6	0.121
Female	37.5	33.2		20.0	35.4	
Age						
≤70	43.8	46.5	0.751	36.0	47.1	0.290
> 70	56.2	53.5		64.0	52.9	
Tumor stage						
T1–2	50.0	30.0	0.009*	44.0	32.5	0.246
T3–4	50.0	70.0		56.0	67.5	
Node stage						
Negative	60.0	53.1	0.395	52.0	54.5	0.811
Positive	40.0	46.9		48.0	45.5	
Metastasis stage						
M0	97.8	91.7	0.147	100	92.1	0.153
M1	2.2	8.3		0	7.9	
Grade						
1–2	93.8	92.2	0.707	88.0	92.9	0.376
3	6.2	7.8		12.0	7.1	
Vascular invasion						
Absent	82.6	75	0.27	84	75.5	0.343
Present	17.4	25		16	24.5	
Perineural invasion						
Absent	84.8	83.8	0.869	92	83.1	0.250
Present	15.2	16.2		8	16.9	
Adjuvant therapy						
No	70.0	69.4	0.945	52.4	71.4	0.072
Yes	30.0	30.6		47.6	28.6	
Neoadjuvant therapy						
No	65.2	80.9	0.021*	68	78	0.205
Yes	34.8	19.1		32	22	
MSH6						
Negative	0	100	0.518	0	100	0.663
Positive	17.3	82.7		8.7	91.3	
PMS2						
Negative	11.1	88.9	0.669	0	100	0.345
Positive	16.5	84.5		9.1	90.9	

*p < 0.05 was considered significant

We then examined the status of the MMR pathway in patient samples by evaluating a possible association of MMR protein expression with MRN combined protein expression. All cases were positive for MLH1 and MSH2 expression; none were classified as MSI-high (MMR-negative). Expression of MSH6 and PMS2 was absent in 0.8% (2/256) and 3.6% (9/252) of cases, respectively, and expression of these proteins was not significantly associated with expression of MRN combined proteins in either TC or TP samples (Table 3).

### Association between the MRN combined expression and survival outcomes

**Fig. 2:**
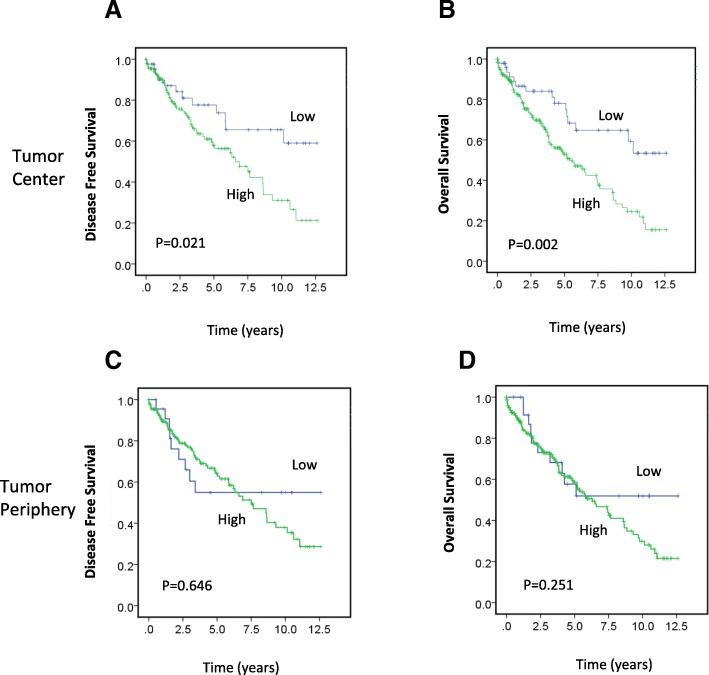
Association between MRN complex protein expression in the TC and TP and survival. **a-d** Kaplan–Meier survival analysis illustrating DFS (**a, c**) and OS (**b, d**) of patients with high (green line) and low (blue line) MRN complex protein expression in the TC (**a, b**) and TP (**c, d**)

Patients with high expression of MRN complex proteins in the TC had significantly worse DFS (*P* = 0.021; Fig. 2a) and OS (*P* = 0.002; Fig. 2b) than patients with low expression. Significant differences in survival were not seen between patients with high or low expression of MRN complex proteins in the TP (DFS, *P* = 0.646, Fig. 2c; OS, *P* = 0.251; Fig. 2d). In addition, we also investigated clinical outcomes of MRE11 and NBS1 protein expression in relation to histological grade tumors. Interestingly, we found that high MRE11 expression was associated with a worse overall survival when patients in the early/low-grade subgroup were analyzed (*P* = 0.045, Additional file 1: Figure S1A). However, a similar significant effect of high MRE11 expression on DFS was not seen in this same patient group (data not shown). No significant relationship between NBS1 expression and OS was identified in early/low-grade tumors (Additional file 1: Figure S1C). In contrast, high NBS1 expression was significantly associated with worse overall survival in high-grade tumors (*P* = 0.045, Additional file 1: Figure S1D), suggesting that both MRE11 and NBS1 may act as potential prognostic indicators in different patient groups.

Using univariate Cox regression analysis, we found that high combined expression of the three proteins in the TC was significantly associated with reduced DFS (hazard ratio [HR] = 2.069, 95% confidence interval [CI] 1.102–3.882, *P* = 0.024; Table 4). Additionally, multivariate Cox analysis demonstrated that MRN complex expression (HR = 2.114, 95% CI 1.096–4.078, *P* = 0.026) and perineural invasion (HR = 2.16, 95% CI 1.209–3.859, *P* = 0.009) remained significantly associated with a worse DFS (Table 4), implying that those markers together are strongly prognostic for DFS in rectal cancer patients.

**Table 4:** Cox regression analyses of combined MRN expression with disease-free survival

	n (%)	Univariate			Multivariate		
		HR	95% CI	*P* Value	HR	95%	*P* Value
MRN TC^a^							
High	81.9	2.069	1.102–3.882	0.024*	2.114	1.096–4.078	0.026*
Low	18.1						
Sex							
Male	66.4	1.041	0.633–1.711	0.876			
Female	33.6						
Age							
≤70	46.0	1.294	0.789–2.125	0.307			
> 70	54.0						
Tumor stage							
T1–2	33.6	1.501	0.897–2.512	0.122			
T3–4	66.4						
Node stage							
Negative	54.3	1.44	0.976–2.126	0.066			
Positive	45.7						
Grade							
1–2	92.5	1.537	0.823–2.872	0.178			
3	7.5						
Vascular invasion							
Absent	76.3	1.167	0.638–2.134	0.617			
Present	23.7						
Perineural invasion							
Absent	84.0	2.334	1.310–4.157	0.004*	2.16	1.209–3.859	0.009*
Present	16.0						
Adjuvant therapy							
No	69.5	0.602	0.341–1.063	0.08			
Yes	30.5						
Neoadjuvant therapy							
No	78.0	0.855	0.529–1.381	0.521			
Yes	22.0						
MRN TC by LN^b^							
LN-negative	54.1				1.339	0.589–3.042	0.486
LN-positive	45.9				3.472	1.051–11.454	0.047*

*HR* hazard ratio, *CI* confidence interval, *TC* tumor center, *LN* lymph node

^a^Three marker combined expression in the tumor center; ^b^ denotes interaction

*p < 0.05 was considered significant

### Correlation of the MRN combined expression with neoadjuvant radiotherapy

**Fig. 3:**
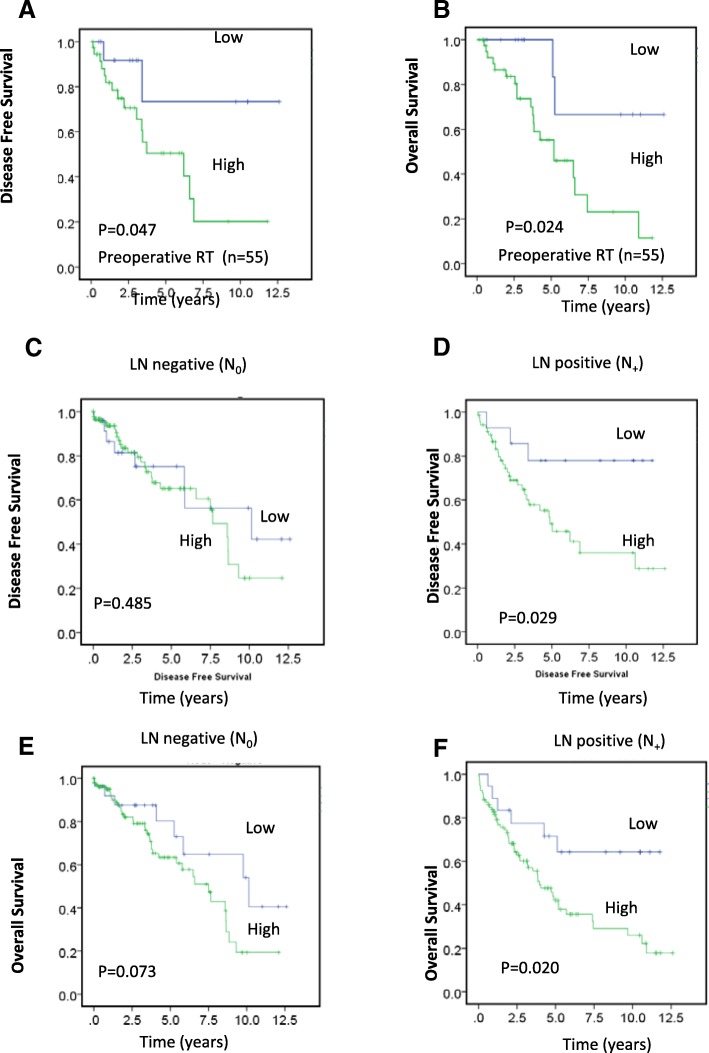
Association between preoperative MRN protein expression in the TC and survival and combined MRN protein expression according to preoperative radiotherapy and LN involvement. **a, b** Kaplan–Meier survival analysis illustrating DFS (**a**) and OS (**b**) in preoperative radiotherapy patient groups with low (blue line) and high (green line) MRN complex panel expression. **c-f** Kaplan–Meier survival analysis illustrating DFS (**c, d**) and OS (**e, f**) in patients with high (green line) and low (blue line) MRN combined expression, in LN-negative (**c, e**) and -positive (**d, f**) rectal cancers

Preoperative or neoadjuvant therapy is the standard treatment for locally advanced rectal cancer [28]. In this study, we examined the possible association between clinicopathological characteristics and survival outcomes with preoperative expression of MRN combined proteins in the 55 patients who had received preoperative radiotherapy. Of 55 patients, 37 (67.3%) were male and 18 (33.6%) were female (Table 1). In the subgroup of patients who received neo-adjuvant radiotherapy, higher combined expression of the MRN complex proteins was significantly associated with worse DFS (Fig. 3a, *P* = 0.024) and OS (Fig. 3b, *P* = 0.028). Multivariate analysis in patients who received preoperative radiotherapy revealed that variables negatively impacting OS included higher histological grade (HR = 7.275, 95% CI 1.842–28.730, *P* = 0.005), high expression of MRN complex proteins (HR = 4.196, 95% CI 0.968–18.191, *P* = 0.045), and male sex (HR = 3.017, 95% CI 1.199–7.592, *P* = 0.019) (Table 5).

**Table 5:** Multivariate analysis of combined MRN expression association with overall survival in preoperative radiotherapy patients

	Multivariate		
	HR	95%	*P* Value
MRN combined TC expression	4.196	0.968–18.191	0.045
Grade	7.275	1.842–28.730	0.005
Sex	3.017	1.199–7.592	0.019

### Prognostic implications of MRN complex proteins in lymph node (LN)-positive subgroup

DFS of rectal cancer patients with high expression of the MRN combined panel was significantly worse than that of patients with low expression. Interestingly, when patients were grouped according to LN involvement, high MRN combined expression was associated with worse DFS and OS in patients with LN-positive tumors (*n* = 119) (DFS, *P* = 0.029, Fig. 3d; OS, *P* = 0.020; Fig. 3f) but not in those with LN-negative tumors (*n* = 140) (DFS, *P* = 0.485, Fig. 3c; OS, *P* = 0.073, Fig. 3e). By multivariate Cox analysis in the LN-positive subgroup, expression of the MRN combined panel in the TC significantly correlated with DFS (HR = 3.474, 95% CI 1.054–11.451, *P* = 0.041) (Table 4). This suggests that the combined expression of the MRN complex proteins may be associated with LN involvement in relation to patient survival, and certainly this needs to be verified in a larger sample set.

## Discussion

Although neoadjuvant chemoradiotherapy has been shown to improve outcomes over surgery alone [5–9], responses are variable between individuals and difficult to predict [10, 11]. Methods for predicting response would enable better treatment decisions to be made. The TRG score, which reflects early response to treatments such as radiotherapy, is significantly associated with late response outcomes including recurrence and survival [8–10]. However, there is a need for accurate, reliable biomarkers of tumor radiosensitivity, to enable better treatment decisions before any therapy is administered. This could avoid unnecessary adverse effects [12, 13] in patients who are unlikely to benefit from radiotherapy.

Efficient DNA damage detection, signaling, and repair after radiotherapy can protect tumor cells against damage. Additionally, avoidance of apoptosis or cell cycle arrest can also allow tumor cells to proliferate even after accumulating DNA damage from radiotherapy. These processes can prolong tumor cell survival and promote poor clinical outcomes. Thus, DNA damage-related proteins are potential biomarkers of tumor radiosensitivity. The ten genes in the RSI are not directly involved in DSB repair, but are often closely connected to DSB repair in functional networks [15]. Of the dozens of molecules studied that might predict survival after preoperative therapy in colorectal cancer patients, many are involved in apoptosis, cell cycle regulation, and DNA damage [14]. Because of its well-known roles in apoptosis and in linking genetic stability to the cell cycle, the association of tumor suppressor p53 with treatment outcomes has been extensively explored; however, these studies have had very inconsistent findings [14]. p21, which is a target of p53, has also been implicated as a potential biomarker. In patients with unresectable rectal cancer treated with preoperative chemoradiotherapy, p21 expression was associated with worse survival, even when adjusted for tumor response [29]. Further studies are required to identify and confirm reliable radiosensitivity biomarkers, but DNA repair proteins remain attractive targets.

Here, we established a biomarker panel comprising the three proteins of the MRN complex, MRE11, RAD50 and NBS1. To compare our previous study that identified the combined expression of MRE11 and ATM as a prognostic biomarker [24], the combined MRN expression had high sensitivity and specificity in samples taken from both the TC and TP. The sensitivity, specificity, and overall accuracy were higher for the combined MRN expression panel than for combined MRE11/ATM expression, both in the TC and TP. We found that high expression of the three MRN complex proteins in the TC was significantly associated with DFS and OS in rectal cancer patients. Rectal cancer patients with high expression of all three MRN proteins are twice as likely to have a poorer DFS (HR = 2.114, 95% CI 1.096–4.078, *P* = 0.026) and four times more likely to have poor OS (HR = 4.196, 95% CI 0.968–18.191, *P* = 0.045) outcomes. Interestingly, none of the other clinicopathologic variables were significantly associated with combined MRN expression. Therefore, this panel appears to be specifically prognostic of DFS and OS. When examining the subset of patients who received preoperative radiotherapy, the association between combined MRN expression and outcome remained significant. It is tempting to hypothesise that the prognostic value of this panel may be related to tumor radiosensitivity, and future research is warranted in a larger definitive cohort.

Interestingly, high MRN protein levels are associated with better outcomes in some other cancer types. In early breast cancer, patients with high MRN complex expression experienced the greatest reduction in recurrence from radiotherapy [30]. In two different studies of bladder cancer patients, high MRE11 expression was associated with better cancer-specific survival in patients who underwent radiotherapy rather than a cystectomy [31, 32]. Therefore, the MRN complex may play a very different role in cancers arising from different tissues. Alternatively, the prognostic value of the MRN complex expression may be dependent on certain combinations of chemotherapy, radiotherapy, and surgery, which vary between the treatment modalities preferred for different cancers.

When patients were classified by LN involvement, the association of combined MRN complex protein expression with DFS and OS was observed in LN-positive patients but not LN-negative patients. The value of analyzing LN involvement to predict outcomes has been established. In a study of rectal cancer patients undergoing long-course neoadjuvant chemoradiotherapy, combining LN involvement with tumor grade was prognostic for survival after treatment [33]. It is feasible that some biomarkers may specifically predict outcomes in patients with LN involvement or those without. Quintanal-Villalonga et al. [34] found that a mutated version of the FGFR4 gene was associated with OS only in LN-involved patients. Our findings suggest that the potential prognostic value of the MRN expression panel may be related to the LN involvement of the patient.

One mechanism that could lead to altered expression of the MRN genes is defective MMR. Giannini et al. [35] found that the *MRE11* gene was mutated in MMR-deficient tumors and cell lines, but not in those with normal MMR function. All of the tumors we tested expressed the two MMR proteins most frequently mutated in MMR-deficient patients, MLH1 and MSH2. The absence of MSH6 or PMS2 protein expression was not significantly associated with combined MRN expression, but this analysis was limited by the very small number of cases lacking expression of either of these proteins. Therefore, the mechanism underlying the prognostic change in MRN expression identified here seems to be independent of the MMR pathway, and is a subject for further study.

The primary limitation of this study was the inability to analyze the relationship of combined MRN expression with tumor regression response. Only 10.6% of patients were classified as responders to radiotherapy, represented by a TRG score of 0 or 1. Because increased MRN protein expression was associated with worse outcomes in rectal cancer patients, reducing MRN protein expression or activity may possibly sensitize tumors to radiotherapy. MRN complex inhibitors, including mirin and telomelysin, have great radiosensitizing effects in preclinical studies [36–38]. Telomelysin is undergoing Phase I and II trials for use in patients with melanoma (NCT03190824), esophageal cancer (NCT03213054), and hepatocellular carcinoma (NCT02293850). The high expression of MRN complex consitituents could be a predictor for poor prognosis and chemoresistance in gastric cancer [39]. An adenovirus targeting RAD50 also showed promise in sensitizing nasopharyngeal carcinoma cells to radiotherapy [40]. Alternatively, in patients with higher MRN expression who are expected to have worse outcomes, additional radiosensitizing treatments could be used in combination with neoadjuvant radiotherapy. Heat treatment, for example, shows good radiosensitizing effects in cells and is being explored in cancer patients [41]. Dynlacht et al. [42] found that heat radiosensitization was dependent on a functioning MRE11 protein, further suggesting the utility of this treatment in high MRN expression tumors.

## Conclusions

The combined expression of the three MRN complex subunits was significantly associated with OS and DFS in rectal cancer patients, including those treated with neoadjuvant radiotherapy. The findings in this study support the proposal that high tissue expression levels of the three MRN complex proteins are prognostic indicators in rectal cancer and in response to preoperative therapy. The association of MRN proteins with radiosensitivity also suggests that the three MRN complex proteins could be targets for the future development of radiosensitizing agents.

## Additional file

Additional file 1: **Figure S1.** Survival outcomes in relation to MRE11 and NBS1 protein expression in rectal cancer tissues. Kaplan-Meier analysis was performed to measure the overall survival (OS) for high (red line) vs. low (blue line) MRE11, NBS1 protein expression in patients with rectal cancer. (A-D) Comparison of survival curves according to histological grade between MRE11 in low-grade (A, G1–2, *n* = 230) and high-grade (B, G3, *n* = 19) subgroups; NBS1 in low vs. high grade (C, D) subgroups, respectively. (PDF 163 kb)

## Abbreviations

CI: Confidence interval
DFS: Disease-free survival
HR: Hazard ratio
LN: Lymph node
MMR: Mismatch repair
MRN: MRE11-RAD50-NBS1
MSI: Microsatellite instability
OS: Overall survival
ROC-AUC: Receiver operator characteristic area under the curve
TC: Tumor center
TP: Tumor periphery at the invasive edge
TRG: Tumor regression grade

## References

[1] Siegel RL, Miller KD, Fedewa SA, Ahnen DJ, Meester RGS, Barzi A, Jemal A (2017). Colorectal cancer statistics, 2017. CA Cancer J Clin.

[2] Jemal A, Bray F, Center MM, Ferlay J, Ward E, Forman D (2011). Global cancer statistics. CA Cancer J Clin.

[3] Tamas K, Walenkamp AM, de Vries EG, van Vugt MA, Beets-Tan RG, van Etten B, de Groot DJ, Hospers GA (2015). Rectal and colon cancer: not just a different anatomic site. Cancer Treat Rev.

[4] Lee YC, Lee YL, Chuang JP, Lee JC (2013). Differences in survival between colon and rectal cancer from SEER data. PLoS One.

[5] Kapiteijn E, Marijnen CA, Nagtegaal ID, Putter H, Steup WH, Wiggers T, Rutten HJ, Pahlman L, Glimelius B, van Krieken JH (2001). Preoperative radiotherapy combined with total mesorectal excision for resectable rectal cancer. N Engl J Med.

[6] Folkesson J, Birgisson H, Pahlman L, Cedermark B, Glimelius B, Gunnarsson U (2005). Swedish rectal Cancer trial: long lasting benefits from radiotherapy on survival and local recurrence rate. J Clin Oncol.

[7] Rahbari NN, Elbers H, Askoxylakis V, Motschall E, Bork U, Buchler MW, Weitz J, Koch M (2013). Neoadjuvant radiotherapy for rectal cancer: meta-analysis of randomized controlled trials. Ann Surg Oncol.

[8] Colorectal Cancer Collaborative Group (2001). Adjuvant radiotherapy for rectal cancer: a systematic overview of 8,507 patients from 22 randomised trials. Lancet.

[9] Camma C, Giunta M, Fiorica F, Pagliaro L, Craxi A, Cottone M (2000). Preoperative radiotherapy for resectable rectal cancer: a meta-analysis. Jama.

[10] Garcia-Aguilar J, Hernandez de Anda E, Sirivongs P, Lee SH, Madoff RD, Rothenberger DA (2003). A pathologic complete response to preoperative chemoradiation is associated with lower local recurrence and improved survival in rectal cancer patients treated by mesorectal excision. Dis Colon Rectum.

[11] Das P, Skibber JM, Rodriguez-Bigas MA, Feig BW, Chang GJ, Wolff RA, Eng C, Krishnan S, Janjan NA, Crane CH (2007). Predictors of tumor response and downstaging in patients who receive preoperative chemoradiation for rectal cancer. Cancer.

[12] Birgisson H, Pahlman L, Gunnarsson U, Glimelius B (2005). Adverse effects of preoperative radiation therapy for rectal cancer: long-term follow-up of the Swedish rectal Cancer trial. J Clin Oncol.

[13] Birgisson H, Pahlman L, Gunnarsson U, Glimelius B (2007). Late adverse effects of radiation therapy for rectal cancer - a systematic overview. Acta Oncol.

[14] Kim NK, Hur H (2015). New perspectives on predictive biomarkers of tumor response and their clinical application in preoperative Chemoradiation therapy for rectal Cancer. Yonsei Med J.

[15] Eschrich S, Zhang H, Zhao H, Boulware D, Lee JH, Bloom G, Torres-Roca JF (2009). Systems biology modeling of the radiation sensitivity network: a biomarker discovery platform. Int J Radiat Oncol Biol Phys.

[16] Ahmed KA, Chinnaiyan P, Fulp WJ, Eschrich S, Torres-Roca JF, Caudell JJ (2015). The radiosensitivity index predicts for overall survival in glioblastoma. Oncotarget.

[17] Foy JP, Bazire L, Ortiz-Cuaran S, Deneuve S, Kielbassa J, Thomas E, Viari A, Puisieux A, Goudot P, Bertolus C (2017). A 13-gene expression-based radioresistance score highlights the heterogeneity in the response to radiation therapy across HPV-negative HNSCC molecular subtypes. BMC Med.

[18] Tian H, Gao Z, Li H, Zhang B, Wang G, Zhang Q, Pei D, Zheng J (2015). DNA damage response--a double-edged sword in cancer prevention and cancer therapy. Cancer Lett.

[19] Harfe BD, Jinks-Robertson S (2000). DNA mismatch repair and genetic instability. Annu Rev Genet.

[20] Thibodeau SN, Bren G, Schaid D (1993). Microsatellite instability in cancer of the proximal colon. Science (New York, NY).

[21] Zhao J, Guo Z, Pei S, Song L, Wang C, Ma J, Jin L, Ma Y, He R, Zhong J (2017). pATM and gammaH2AX are effective radiation biomarkers in assessing the radiosensitivity of 12C6+ in human tumor cells. Cancer Cell Int.

[22] van den Bosch M, Bree RT, Lowndes NF (2003). The MRN complex: coordinating and mediating the response to broken chromosomes. EMBO Rep.

[23] Lavin MF, Kozlov S, Gatei M, Kijas AW (2015). ATM-dependent phosphorylation of all three members of the MRN complex: from sensor to adaptor. Biomolecules.

[24] Ho V, Chung L, Revoltar M, Lim SH, Tut TG, Abubakar A, Henderson CJ, Chua W, Ng W, Lee M (2016). MRE11 and ATM expression levels predict rectal Cancer survival and their association with radiotherapy response. PLoS One.

[25] Ho V, Chung L, Singh A, Lea V, Revoltar M, Lim SH, Tut TG, Ng W, Lee M, de Souza P, et al. Early Postoperative Low Expression of RAD50 in Rectal Cancer Patients Associates with Disease-Free Survival. Cancers. 2017;9(12) 10.3390/cancers9120163. 10.3390/cancers9120163 PMC5742811 29189711

[26] Edge SB, Compton CC (2010). The American joint committee on Cancer: the 7th edition of the AJCC cancer staging manual and the future of TNM. Ann Surg Oncol.

[27] Chung L, Moore K, Phillips L, Boyle FM, Marsh DJ, Baxter RC (2014). Novel serum protein biomarker panel revealed by mass spectrometry and its prognostic value in breast cancer. Breast Cancer Res : BCR.

[28] Dayde D, Tanaka I, Jain R, Tai MC, Taguchi A. Predictive and Prognostic Molecular Biomarkers for Response to Neoadjuvant Chemoradiation in Rectal Cancer. Int J Mol Sci. 2017;18(3) 10.3390/ijms18030573. 10.3390/ijms18030573 PMC5372589 28272347

[29] Reerink O, Karrenbeld A, Plukker JT, Verschueren RC, Szabo BG, Sluiter WJ, Hospers GA, Mulder NH (2004). Molecular prognostic factors in locally irresectable rectal cancer treated preoperatively by chemo-radiotherapy. Anticancer Res.

[30] Soderlund K, Stal O, Skoog L, Rutqvist LE, Nordenskjold B, Askmalm MS (2007). Intact Mre11/Rad50/Nbs1 complex predicts good response to radiotherapy in early breast cancer. Int J Radiat Oncol Biol Phys.

[31] Choudhury A, Nelson LD, Teo MT, Chilka S, Bhattarai S, Johnston CF, Elliott F, Lowery J, Taylor CF, Churchman M (2010). MRE11 expression is predictive of cause-specific survival following radical radiotherapy for muscle-invasive bladder cancer. Cancer Res.

[32] Laurberg JR, Brems-Eskildsen AS, Nordentoft I, Fristrup N, Schepeler T, Ulhoi BP, Agerbaek M, Hartmann A, Bertz S, Wittlinger M (2012). Expression of TIP60 (tat-interactive protein) and MRE11 (meiotic recombination 11 homolog) predict treatment-specific outcome of localised invasive bladder cancer. BJU Int.

[33] Lindebjerg J, Spindler KL, Ploen J, Jakobsen A (2009). The prognostic value of lymph node metastases and tumour regression grade in rectal cancer patients treated with long-course preoperative chemoradiotherapy. Colorectal Dis.

[34] Quintanal-Villalonga A, Carranza-Carranza A, Melendez R, Ferrer I, Molina-Pinelo S, Paz-Ares L (2017). Prognostic Role of the FGFR4-388Arg Variant in Lung Squamous-Cell Carcinoma Patients With Lymph Node Involvement. Clin Lung Cancer.

[35] Giannini G, Ristori E, Cerignoli F, Rinaldi C, Zani M, Viel A, Ottini L, Crescenzi M, Martinotti S, Bignami M (2002). Human MRE11 is inactivated in mismatch repair-deficient cancers. EMBO Rep.

[36] Dupre A, Boyer-Chatenet L, Sattler RM, Modi AP, Lee JH, Nicolette ML, Kopelovich L, Jasin M, Baer R, Paull TT (2008). A forward chemical genetic screen reveals an inhibitor of the Mre11-Rad50-Nbs1 complex. Nat Chem Biol.

[37] Garner KM, Pletnev AA, Eastman A (2009). Corrected structure of mirin, a small-molecule inhibitor of the Mre11-Rad50-Nbs1 complex. Nat Chem Biol.

[38] Kuroda S, Urata Y, Fujiwara T (2012). Ataxia-telangiectasia mutated and the Mre11-Rad50-NBS1 complex: promising targets for radiosensitization. Acta Med Okayama.

[39] Altan B, Yokobori T, Ide M, Bai T, Yanoma T, Kimura A, Kogure N, Suzuki M, Bao P, Mochiki E (2016). High expression of MRE11-RAD50-NBS1 is associated with poor prognosis and Chemoresistance in gastric Cancer. Anticancer Res.

[40] Chang L, Huang J, Wang K, Li J, Yan R, Zhu L, Ye J, Wu X, Zhuang S, Li D (2016). Targeting Rad50 sensitizes human nasopharyngeal carcinoma cells to radiotherapy. BMC Cancer.

[41] Dewey WC (2009). Arrhenius relationships from the molecule and cell to the clinic. Int J Hyperthermia.

[42] Dynlacht JR, Batuello CN, Lopez JT, Kim KK, Turchi JJ (2011). Identification of Mre11 as a target for heat radiosensitization. Radiat Res.

