# Supplementary material for: Overexpression of the MRE11-RAD50-NBS1 (MRN) complex in rectal cancer correlates with poor response to neoadjuvant radiotherapy and prognosis
Source: BMC Cancer. 2018 Sep 3;18:869. doi: 10.1186/s12885-018-4776-9 (PMC6122630; doi:10.1186/s12885-018-4776-9)
Supplement: Supplementary file 1 — Figure S1. Survival outcomes in relation to MRE11 and NBS1 protein expression in rectal cancer tissues. Kaplan-Meier analysis was performed to measure the overall survival (OS) for high (red line) vs. low (blue line) MRE11, NBS1 protein expression in patients with rectal cancer. (A-D) Comparison of survival curves according to histological grade between MRE11 in low-grade (A, G1–2, n = 230) and high-grade (B, G3, n = 19) subgroups; NBS1 in low vs. high grade (C, D) subgroups, respectively. (PDF 163 kb) [file 12885_2018_4776_MOESM1_ESM.pdf]

Figure S1

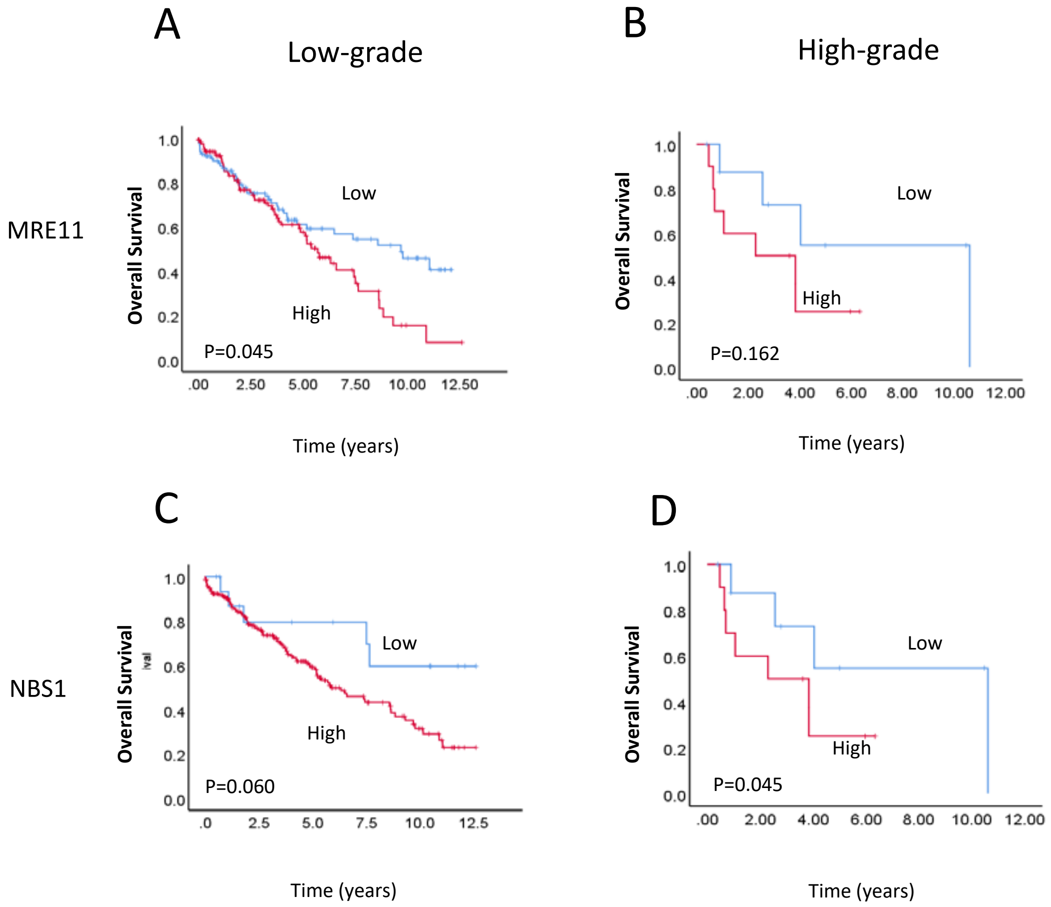

Figure S1. Survival outcomes in relation to MRE11 and NBS1 protein expression in rectal cancer tissues. Kaplan-Meier analysis was performed to measure the overall survival (OS) for high (red line) *vs.* low (blue line) MRE11, NBS1 protein expression in patients with rectal cancer. (A-D) Comparison of survival curves according to histological grade between MRE11 in low-grade (A, G1-2, n=230) and high-grade (B, G3, n=19) subgroups; NBS1 in low *vs.* high grade (C, D) subgroups, respectively.
